# Supplementary material for: Formal and informal care received by middle-aged and older adults with chronic conditions in Canada: CLSA data
Source: PLoS One. 2020 Jul 7;15(7):e0235774. doi: 10.1371/journal.pone.0235774 (PMC7340302; doi:10.1371/journal.pone.0235774)
Supplement: S1 Table — aCLSA indicates Canadian Longitudinal Study on Aging. (DOCX) [file pone.0235774.s001.docx]

**S1 Table. Chronic condition classification based on different methods (step one)**

| **Chronic condition** | **Classification in step one** | **Griffith et al.** [1] | **CLSA^a^ questionnaire** [2] | **CLSA diseases based on ICD-10** [3] |
| --- | --- | --- | --- | --- |
| Asthma | Respiratory | Respiratory | Respiratory | Respiratory system |
| Emphysema, chronic bronchitis, chronic obstructive pulmonary disease (COPD), or chronic changes in lungs due to smoking |  |  |  |  |
| Cataracts | Ophthalmologic | Ophthalmologic | Vision | Eye and adnexa |
| Glaucoma |  |  |  |  |
| Macular degeneration |  |  |  |  |
| Cancer | Cancer | Cancer | Cancer | Neoplasms |
| Under-active thyroid gland | Endocrine/Metabolic | Endocrine/Metabolic | Other conditions | Endocrine, nutritional and metabolic diseases |
| Over-active thyroid gland |  |  |  |  |
| Diabetes, borderline diabetes or high blood sugar |  |  | Cardiac/Cardiovascular |  |
| High blood pressure or hypertension | Circulatory | Vascular |  | Circulatory system |
| Peripheral vascular disease or poor circulation in limbs |  |  |  |  |
| Heart disease (including congestive heart failure (CHF)) |  | Cardiac |  |  |
| Heart attack or myocardial infarction |  |  |  |  |
| Angina (or chest pain due to heart disease) |  |  |  |  |
| Mini-stroke or TIA (Transient Ischemic Attack) |  | Neurological |  |  |
| Stroke or CVA (cerebrovascular accident) |  |  |  |  |
| Multiple sclerosis | Neurological |  | Neurological | Nervous system |
| Parkinsonism or Parkinson's Disease |  |  |  |  |
| Epilepsy |  |  |  |  |
| Migraine headaches |  |  |  |  |
| Memory problems |  | Not considered |  | Cognition, perception, emotional state and behaviour |
| Dementia or Alzheimer’s disease |  | Psychiatric |  | Nervous system or diseases without precise specification |
| Anxiety disorder | Mental |  | Mental Health | Mental and bahavioural disorders |
| Mood disorder |  |  |  |  |
| Back problems excluding fibromyalgia and arthritis | Musculoskeletal | Other risk factors/symptoms | Other conditions | Musculoskeletal system and connective tissue |
| Osteoporosis |  | Musculoskeletal | Other conditions |  |
| Osteoarthritis in the knee |  |  | Osteoarthritis |  |
| Osteoarthritis in one or both hands |  |  |  |  |
| Osteoarthritis in one or both hips |  |  |  |  |
| Rheumatoid arthritis |  |  | Arthritis |  |
| Other type of arthritis |  | Not considered |  |  |
| Intestinal or stomach ulcers | Gastrointestinal | Gastrointestinal | Gastrointestinal | Digestive system |
| Bowel disorder |  |  |  |  |
| Bowel incontinence |  |  |  |  |
| Urinary incontinence | Genitourinary | Genitourinary |  | Genitourinary system |
| Kidney disease or kidney failure |  | Renal | Other conditions |  |

^a^CLSA indicates Canadian Longitudinal Study on Aging

**References**

1. Griffith L. The Impact of Chronic Condition List on Prevalence and the Relationship between MCCs and Disability, Social Participation, and Self-Rated Health: Data from the Canadian Longitudinal Study on Aging. 2018. https://www.clsa-elcv.ca/sites/default/files/presentations/griffith_cag_multimorbidity_2018_1.pdf.

2. CLSA. Data Support Documentation for the Canadian Longitudinal Study on Aging (CLSA). 2018. https://www.clsa-elcv.ca/researchers/data-support-documentation. Accessed 7 May 2019.

3. CLSA. Data Preview Portal for the Canadian Longitudinal Study on Aging (CLSA). 2018. https://datapreview.clsa-elcv.ca/. Accessed 24 Aug 2018.
