# Supplementary material for: Formal and informal care received by middle-aged and older adults with chronic conditions in Canada: CLSA data
Source: PLoS One. 2020 Jul 7;15(7):e0235774. doi: 10.1371/journal.pone.0235774 (PMC7340302; doi:10.1371/journal.pone.0235774)
Supplement: S2 Table — Bolded text indicates a chronic condition that was considered as a stand-alone classification. (DOCX) [file pone.0235774.s002.docx]

**S2 Table. Chronic condition classification by comparing the total number of hours of formal and informal care among chronic conditions**

| **Chronic condition** | **Classification in step one from S1 Table** | **Step two** | | **Step three** | |
| --- | --- | --- | --- | --- | --- |
|  |  | **Total number of hours - formal and informal hours** | **Classification after this step (total hours >120 hours)** | **Difference from the average total hours within the classification** | **Classification after this step (difference over ± 30 hours** |
| Asthma | Respiratory | 35.35 | Respiratory | -21.46 | Respiratory |
| Emphysema, chronic bronchitis, chronic obstructive pulmonary disease (COPD), or chronic changes in lungs due to smoking | Respiratory | 78.27 | Respiratory | 21.46 | Respiratory |
| Cataracts | Ophthalmologic | 43.32 | Ophthalmologic | -6.01 | Ophthalmologic |
| Glaucoma | Ophthalmologic | 41.30 | Ophthalmologic | -8.04 | Ophthalmologic |
| Macular degeneration | Ophthalmologic | 63.38 | Ophthalmologic | 14.05 | Ophthalmologic |
| Cancer | Cancer | 43.67 | Cancer | 0.00 | Cancer |
| Under-active thyroid gland | Endocrine/Metabolic | 42.68 | Endocrine/Metabolic | 1.52 | Endocrine/Metabolic |
| Over-active thyroid gland | Endocrine/Metabolic | 36.89 | Endocrine/Metabolic | -4.27 | Endocrine/Metabolic |
| Diabetes, borderline diabetes or high blood sugar | Endocrine/Metabolic | 43.91 | Endocrine/Metabolic | 2.75 | Endocrine/Metabolic |
| High blood pressure (HBP) or hypertension | Circulatory | 33.86 | Circulatory | **-31.32** | **Hypertension** |
| Peripheral vascular disease or poor circulation in limbs | Circulatory | 77.77 | Circulatory | 12.59 | Circulatory |
| Heart disease (including congestive heart failure, or CHF) | Circulatory | 53.71 | Circulatory | -11.48 | Circulatory |
| Heart attack or myocardial infarction | Circulatory | 67.63 | Circulatory | 2.45 | Circulatory |
| Angina (or chest pain due to heart disease) | Circulatory | 77.86 | Circulatory | 12.67 | Circulatory |
| Mini-stroke or TIA (Transient Ischemic Attack) | Circulatory | 80.27 | Circulatory | 15.09 | Circulatory |
| Stroke or CVA (cerebrovascular accident) | Circulatory | **120.87** | **Stroke** | 0.00 | **Stroke** |
| Multiple sclerosis (MS) | Neurological | **251.78** | **Multiple sclerosis** | 0.00 | **Multiple sclerosis** |
| Parkinsonism or Parkinson's Disease | Neurological | **192.25** | **Parkinsonism** | 0.00 | **Parkinsonism** |
| Epilepsy | Neurological | 78.85 | Neurological | 18.63 | Neurological |
| Migraine headaches | Neurological | 41.58 | Neurological | -18.63 | Neurological |
| Memory problems | Neurological | **170.24** | **Memory problems** | 0.00 | **Memory problems** |
| Dementia or Alzheimer’s disease | Neurological | **250.21** | **Dementia** | 0.00 | **Dementia** |
| Anxiety disorder | Mental | 56.30 | Mental | 2.22 | Mental |
| Mood disorder | Mental | 51.87 | Mental | -2.22 | Mental |
| Back problems excluding fibromyalgia and arthritis | Musculoskeletal | 41.27 | Musculoskeletal | -11.38 | Musculoskeletal |
| Osteoporosis | Musculoskeletal | 61.44 | Musculoskeletal | 8.80 | Musculoskeletal |
| Osteoarthritis in the knee | Musculoskeletal | 47.84 | Musculoskeletal | -4.80 | Musculoskeletal |
| Osteoarthritis in one or both hands | Musculoskeletal | 55.02 | Musculoskeletal | 2.38 | Musculoskeletal |
| Osteoarthritis in one or both hips | Musculoskeletal | 66.81 | Musculoskeletal | 14.17 | Musculoskeletal |
| Rheumatoid arthritis | Musculoskeletal | 50.71 | Musculoskeletal | -1.93 | Musculoskeletal |
| Other type of arthritis | Musculoskeletal | 45.41 | Musculoskeletal | -7.23 | Musculoskeletal |
| Intestinal or stomach ulcers | Gastrointestinal | 40.33 | Gastrointestinal | -4.02 | Gastrointestinal |
| Bowel disorder | Gastrointestinal | 48.38 | Gastrointestinal | 4.02 | Gastrointestinal |
| Bowel incontinence | Gastrointestinal | **138.78** | **Bowel incontinence** | 0.00 | **Bowel incontinence** |
| Urinary incontinence | Genitourinary | 78.84 | Genitourinary | 4.35 | Genitourinary |
| Kidney disease or kidney failure | Genitourinary | 70.14 | Genitourinary | -4.35 | Genitourinary |
| None of above |  | 2.38 |  | 0.00 |  |

Bolded text indicates a chronic condition that was considered as a stand-alone classification
