# Supplementary material for: Formal and informal care received by middle-aged and older adults with chronic conditions in Canada: CLSA data
Source: PLoS One. 2020 Jul 7;15(7):e0235774. doi: 10.1371/journal.pone.0235774 (PMC7340302; doi:10.1371/journal.pone.0235774)
Supplement: S3 Table — Bolded text indicates a chronic condition that was considered as a stand-alone classification. (DOCX) [file pone.0235774.s003.docx]

**S3 Table. Final chronic condition classification**

| **Chronic condition** | **Classification in step one from S1 Table** | **Final classification in step three from S2 Table** | **All** | | **Women** | | **Men** | |
| --- | --- | --- | --- | --- | --- | --- | --- | --- |
|  |  |  | **Total number of hours - formal and informal care** | **Difference from the average total hours within the classification** | **Total number of hours - formal and informal care** | **Difference from the average total hours within the classification** | **Total number of hours - formal and informal care** | **Difference from the average total hours within the classification** |
| Asthma | Respiratory | Respiratory | 35.35 | -21.46 | 40.28 | -27.81 | 28.46 | -14.22 |
| Emphysema, chronic bronchitis, chronic obstructive pulmonary disease (COPD), or chronic changes in lungs due to smoking | Respiratory | Respiratory | 78.27 | 21.46 | 95.91 | 27.81 | 56.89 | 14.22 |
| Cataracts | Ophthalmologic | Ophthalmologic | 43.32 | -6.01 | 50.64 | -8.2 | 33.17 | -4.43 |
| Glaucoma | Ophthalmologic | Ophthalmologic | 41.3 | -8.04 | 58.92 | 0.08 | 20.69 | -16.91 |
| Macular degeneration | Ophthalmologic | Ophthalmologic | 63.38 | 14.05 | 66.96 | 8.12 | 58.92 | 21.33 |
| Cancer | Cancer | Cancer | 43.67 | 0 | 49.79 | 0 | 36.37 | 0 |
| Under-active thyroid gland | Endocrine/Metabolic | Endocrine/Metabolic | 42.68 | 1.52 | 46.84 | -2.04 | 28.04 | 2.83 |
| Over-active thyroid gland | Endocrine/Metabolic | Endocrine/Metabolic | 36.89 | -4.27 | 42.95 | -5.93 | 14.42 | -10.79 |
| Diabetes, borderline diabetes or high blood sugar | Endocrine/Metabolic | Endocrine/Metabolic | 43.91 | 2.75 | 56.85 | 7.97 | 33.18 | 7.97 |
| **High blood pressure (HBP) or hypertension** | **Circulatory** | **Hypertension** | **33.86** | **0** | **45.73** | **0** | **22.77** | **0** |
| Peripheral vascular disease or poor circulation in limbs | Circulatory | Cardiac | 77.77 | 6.33 | 76.01 | -15.5 | 79.86 | 19.45 |
| Heart disease (including congestive heart failure, or CHF) | Circulatory | Cardiac | 53.71 | -17.74 | 76.27 | -15.24 | 40.39 | -20.02 |
| Heart attack or myocardial infarction | Circulatory | Cardiac | 67.63 | -3.82 | 105.94 | 14.42 | 53.4 | -7.01 |
| Angina (or chest pain due to heart disease) | Circulatory | Cardiac | 77.86 | 6.41 | 105.35 | 13.84 | 61.17 | 0.76 |
| Mini-stroke or TIA (Transient Ischemic Attack) | Circulatory | Cardiac | 80.27 | 8.82 | 94 | 2.48 | 67.23 | 6.82 |
| **Stroke or CVA (cerebrovascular accident)** | **Circulatory** | **Stroke** | **120.87** | **0** | **97.48** | **0** | **137.93** | **0** |
| **Multiple sclerosis (MS)** | **Neurological** | **Multiple sclerosis** | **251.78** | **0** | **183.48** | **0** | **431.39** | **0** |
| **Parkinsonism or Parkinson's Disease** | **Neurological** | **Parkinsonism** | **192.25** | **0** | **219.36** | **0** | **176.91** | **0** |
| Epilepsy | Neurological | Neurological | 78.85 | 18.63 | 87.77 | 21.26 | 69.81 | 19.03 |
| Migraine headaches | Neurological | Neurological | 41.58 | -18.63 | 45.24 | -21.26 | 31.75 | -19.03 |
| **Memory problems** | **Neurological** | **Memory problems** | **170.24** | **0** | **179.8** | **0** | **161.26** | **0** |
| **Dementia or Alzheimer’s disease** | **Neurological** | **Dementia** | **250.21** | **0** | **251.64** | **0** | **249.23** | **0** |
| Anxiety disorder | Mental | Mental | 56.3 | 2.22 | 69.67 | 7.24 | 34 | -6.2 |
| Mood disorder | Mental | Mental | 51.87 | -2.22 | 55.18 | -7.24 | 46.39 | 6.2 |
| Back problems excluding fibromyalgia and arthritis | Musculoskeletal | Musculoskeletal | 41.27 | -11.38 | 60.04 | -2.42 | 23.08 | -13.68 |
| Osteoporosis | Musculoskeletal | Musculoskeletal | 61.44 | 8.8 | 64.03 | 1.57 | 45.25 | 8.5 |
| Osteoarthritis in the knee | Musculoskeletal | Musculoskeletal | 47.84 | -4.8 | 55.6 | -6.86 | 36.68 | -0.07 |
| Osteoarthritis in one or both hands | Musculoskeletal | Musculoskeletal | 55.02 | 2.38 | 60.43 | -2.03 | 43.14 | 6.39 |
| Osteoarthritis in one or both hips | Musculoskeletal | Musculoskeletal | 66.81 | 14.17 | 80.41 | 17.95 | 42.12 | 5.36 |
| Rheumatoid arthritis | Musculoskeletal | Musculoskeletal | 50.71 | -1.93 | 62.72 | 0.26 | 32.63 | -4.13 |
| Other type of arthritis | Musculoskeletal | Musculoskeletal | 45.41 | -7.23 | 53.98 | -8.48 | 34.38 | -2.38 |
| Intestinal or stomach ulcers | Gastrointestinal | Gastrointestinal | 40.33 | -4.02 | 51.37 | -3.16 | 29.06 | -0.02 |
| Bowel disorder | Gastrointestinal | Gastrointestinal | 48.38 | 4.02 | 57.69 | 3.16 | 29.1 | 0.02 |
| **Bowel incontinence** | **Gastrointestinal** | **Bowel incontinence** | **138.78** | **0** | **122.34** | **0** | **170.73** | **0** |
| Urinary incontinence | Genitourinary | Genitourinary | 78.84 | 4.35 | 74.27 | 0.82 | 92.5 | 12.29 |
| Kidney disease or kidney failure | Genitourinary | Genitourinary | 70.14 | -4.35 | 72.64 | -0.82 | 67.92 | -12.29 |
| None of above |  |  | 2.38 | 0 | 3.14 | 0 | 1.86 | 0 |

Bolded text indicates a chronic condition that was considered as a stand-alone classification
