# Supplementary material for: Formal and informal care received by middle-aged and older adults with chronic conditions in Canada: CLSA data
Source: PLoS One. 2020 Jul 7;15(7):e0235774. doi: 10.1371/journal.pone.0235774 (PMC7340302; doi:10.1371/journal.pone.0235774)
Supplement: S4 Table — aSE indicates standard error. bIndependent samples t test for continuous variables; Chi-squared test for categorical variables. Reported mean, SE, and % were estimated using the pooled (trimmed) inflation weights and the geographic strata variables. (DOCX) [file pone.0235774.s004.docx]

**S4 Table. Full characteristics of study population**

| **Characteristics** | **All** | **Women** | **Men** | **P-value^b^** |
| --- | --- | --- | --- | --- |
|  | **Mean (SE^a^) or Number (%)** | **Mean (SE) or Number (%)** | **Mean (SE) or Number (%)** |  |
| Age (years) | 60.09 (0.07) | 60.24 (0.10) | 59.92 (0.10) | 0.028 |
| 45-54 | 12404 (38.3%) | 6367 (37.7%) | 6037 (39.0%) | 0.002 |
| 55-64 | 14984 (31.2%) | 7673 (31.1%) | 7311 (31.3%) |  |
| 65-74 | 10645 (18.8%) | 5269 (18.8%) | 5376 (18.9%) |  |
| 75+ | 8076 (11.6%) | 3897 (12.5%) | 4179 (10.8%) |  |
| Married/living with a partner in a common-law relationship | 32063 (75.5%) | 14131 (69.3%) | 17932 (81.8%) | < 0.001 |
| White (ethnicity) | 44483 (96.0%) | 22523 (96.6%) | 21960 (95.4%) | < 0.001 |
| Education attainment |  |  |  |  |
| Less than secondary school graduation | 3076 (6.9%) | 1579 (7.1%) | 1497 (6.6%) | < 0.001 |
| Secondary school graduation, no post-secondary education | 5109 (12.5%) | 2795 (13.7%) | 2314 (11.3%) |  |
| Some post-secondary education | 3476 (7.6%) | 1776 (7.9%) | 1700 (7.4%) |  |
| Post-secondary degree/diploma | 34448 (73.0%) | 17056 (71.3%) | 17392 (74.7%) |  |
| Number of people living in household (excluding the participant) | 1.42 (0.01) | 1.32 (0.01) | 1.53 (0.01) | < 0.001 |
| Own dwelling | 39518 (87.7%) | 19519 (86.5%) | 19999 (88.9%) | < 0.001 |
| Household income |  |  |  |  |
| Less than $20,000 | 2366 (4.5%) | 1526 (5.6%) | 840 (3.3%) | < 0.001 |
| $20,000 or more, but less than $50,000 | 10646 (21.6%) | 6137 (24.5%) | 4509 (18.7%) |  |
| $50,000 or more, but less than $100,000 | 15624 (34.5%) | 7466 (33.1%) | 8158 (35.9%) |  |
| $100,000 or more, but less than $150,000 | 8111 (18.8%) | 3568 (17.3%) | 4543 (20.4%) |  |
| $150,000 or more | 6581 (15.7%) | 2720 (13.1%) | 3861 (18.4%) |  |
| Don't know/No answer/Refused | 2781 (4.9%) | 1789 (6.3%) | 992 (3.4%) |  |
| Body Mass Index classification |  |  |  |  |
| Obesity | 12656 (25.7%) | 6377 (25.6%) | 6279 (25.7%) | < 0.001 |
| Overweight | 18838 (40.5%) | 7978 (33.1%) | 10860 (48.1%) |  |
| Normal or underweight | 14615 (33.8%) | 8851 (41.3%) | 5764 (26.2%) |  |
| Type of smoker |  |  |  |  |
| Current smoker | 4824 (11.5%) | 2315 (11.0%) | 2509 (12.0%) | < 0.001 |
| Former smoker | 27948 (58.9%) | 13137 (55.5%) | 14811 (62.3%) |  |
| Never smoked | 13337 (29.6%) | 7754 (33.5%) | 5583 (25.6%) |  |
| Type of drinker |  |  |  |  |
| Regular drinker (at least once a month) | 34160 (75.1%) | 16032 (70.1%) | 18128 (80.2%) | < 0.001 |
| Occasional drinker | 6415 (13.9%) | 4209 (18.2%) | 2206 (9.4%) |  |
| Not a drinker (did not drink in the last 12 months) | 5534 (11.1%) | 2965 (11.7%) | 2569 (10.4%) |  |
| Care received |  |  |  |  |
| No home care received | 39935 (86.9%) | 19507 (84.4%) | 20428 (89.5%) | < 0.001 |
| Formal home care only | 1032 (1.9%) | 587 (2.1%) | 445 (1.7%) |  |
| Informal home care only | 4046 (8.8%) | 2427 (10.5%) | 1619 (7.0%) |  |
| Both formal and informal home care | 1096 (2.4%) | 685 (3.0%) | 411 (1.9%) |  |
| Number of formal care hours among the entire population | 5.74 (0.97) | 7.13 (1.77) | 4.30 (0.74) | 0.142 |
| by age groups |  |  |  |  |
| 45-54 | 5.48 (2.26) | 7.51 (4.36) | 3.46 (1.21) |  |
| 55-64 | 3.97 (0.86) | 4.52 (1.24) | 3.40 (1.18) |  |
| 65-74 | 5.22 (1.41) | 7.43 (2.71) | 2.96 (0.62) |  |
| 75+ | 12.16 (1.92) | 12.03 (1.41) | 12.31 (3.86) |  |
| Number of formal care hours among those who received care | 132.03 (21.84) | 139.78 (34.02) | 120.63 (20.10) | 0.628 |
| Number of informal care hours among the entire population | 24.44 (2.68) | 31.35 (4.02) | 17.34 (3.51) | 0.009 |
| by age groups |  |  |  |  |
| 45-54 | 23.67 (5.02) | 27.13 (5.49) | 20.23 (8.39) |  |
| 55-64 | 19.92 (3.27) | 26.07 (6.09) | 13.63 (2.21) |  |
| 65-74 | 29.01 (7.40) | 43.37 (13.97) | 14.34 (4.41) |  |
| 75+ | 31.69 (5.88) | 39.10 (9.59) | 22.88 (5.96) |  |
| Number of informal care hours among those who received care | 218.75 (23.30) | 232.95 (28.98) | 196.49 (38.91) | 0.452 |
| Chronic condition classification |  |  |  |  |
| Bowel incontinence | 896 (1.8%) | 586 (2.3%) | 310 (1.3%) | < 0.001 |
| Cancer | 7020 (13.1%) | 3612 (14.1%) | 3408 (11.9%) | < 0.001 |
| Cardiac | 8751 (16.8%) | 3724 (14.9%) | 5027 (18.7%) | < 0.001 |
| Endocrine/Metabolic | 12895 (24.7%) | 7373 (28.7%) | 5522 (20.6%) | < 0.001 |
| Gastrointestinal | 6940 (13.7%) | 4071 (15.8%) | 2869 (11.6%) | < 0.001 |
| Genitourinary | 4790 (9.5%) | 3117 (12.6%) | 1673 (6.3%) | < 0.001 |
| Hypertension | 16993 (33.6%) | 8099 (32.4%) | 8894 (35.0%) | < 0.001 |
| Memory problems | 734 (1.7%) | 353 (1.6%) | 381 (1.7%) | 0.560 |
| Mental | 8932 (19.2%) | 5580 (23.5%) | 3352 (14.8%) | < 0.001 |
| Multiple sclerosis | 278 (0.6%) | 200 (0.8%) | 78 (0.3%) | < 0.001 |
| Musculoskeletal | 24503 (50.4%) | 13719 (55.6%) | 10784 (45.1%) | < 0.001 |
| Neurological | 6368 (14.7%) | 4603 (20.9%) | 1765 (8.3%) | < 0.001 |
| Ophthalmologic | 13781 (23.1%) | 7514 (26.4%) | 6267 (19.8%) | < 0.001 |
| Parkinsonism | 171 (0.3%) | 58 (0.2%) | 113 (0.5%) | 0.006 |
| Respiratory | 7436 (15.1%) | 4221 (16.7%) | 3215 (13.4%) | < 0.001 |
| Stroke | 743 (1.5%) | 299 (1.1%) | 444 (1.8%) | < 0.001 |

^a^SE indicates standard error.

^b^Independent samples *t* test for continuous variables; Chi-squared test for categorical variables.

Reported mean, SE, and % were estimated using the pooled (trimmed) inflation weights and the geographic strata variables.
