# Supplementary material for: Formal and informal care received by middle-aged and older adults with chronic conditions in Canada: CLSA data
Source: PLoS One. 2020 Jul 7;15(7):e0235774. doi: 10.1371/journal.pone.0235774 (PMC7340302; doi:10.1371/journal.pone.0235774)
Supplement: S5 Table — (DOCX) [file pone.0235774.s005.docx]

**S5 Table. Formal care and informal care by chronic conditions**

| **Chronic condition** | **Status** | **Formal care** | | | **Informal care** | | |
| --- | --- | --- | --- | --- | --- | --- | --- |
|  |  | **% receiving care** | **Number of hours among those receiving care** | **Overall number of hours** | **% receiving care** | **Number of hours among those receiving care** | **Overall number of hours** |
| **All** |  |  |  |  |  |  |  |
| Bowel incontinence | Yes | 16.3% | 166.05 (40.25) | 27.15 (7.09) | 26.0% | 391.01 (104.98) | 101.74 (28.33) |
| Bowel incontinence | No | 3.6% | 120.75 (13.11) | 4.37 (0.49) | 10.4% | 173.21 (11.31) | 18.06 (1.21) |
| Cancer | Yes | 7.7% | 81.10 (9.41) | 6.26 (0.77) | 16.5% | 218.32 (28.41) | 36.13 (4.84) |
| Cancer | No | 3.3% | 139.14 (16.52) | 4.55 (0.56) | 9.8% | 173.56 (12.84) | 17.06 (1.30) |
| Cardiac | Yes | 9.4% | 124.34 (14.90) | 11.66 (1.46) | 17.8% | 240.96 (25.21) | 42.89 (4.63) |
| Cardiac | No | 2.8% | 123.98 (18.10) | 3.47 (0.52) | 9.4% | 161.55 (13.13) | 15.12 (1.26) |
| Endocrine/Metabolic | Yes | 6.3% | 111.29 (12.19) | 6.97 (0.80) | 14.3% | 224.32 (25.15) | 32.16 (3.71) |
| Endocrine/Metabolic | No | 3.0% | 133.35 (19.62) | 4.01 (0.60) | 9.4% | 160.55 (12.06) | 15.16 (1.17) |
| Gastrointestinal | Yes | 6.7% | 112.44 (12.85) | 7.52 (0.92) | 16.5% | 218.47 (21.06) | 36.15 (3.64) |
| Gastrointestinal | No | 3.4% | 128.02 (16.11) | 4.31 (0.56) | 9.7% | 172.19 (13.85) | 16.73 (1.38) |
| Genitourinary | Yes | 10.7% | 206.26 (42.50) | 21.98 (4.67) | 20.7% | 248.78 (22.97) | 51.55 (5.01) |
| Genitourinary | No | 3.2% | 96.15 (8.23) | 3.03 (0.27) | 9.7% | 168.20 (13.38) | 16.29 (1.33) |
| Hypertension | Yes | 5.9% | 98.11 (9.05) | 5.83 (0.57) | 13.4% | 202.48 (15.07) | 27.07 (2.09) |
| Hypertension | No | 2.8% | 151.08 (23.63) | 4.24 (0.68) | 9.4% | 168.53 (16.94) | 15.82 (1.63) |
| Memory problems | Yes | 15.8% | 384.17 (133.32) | 60.72 (22.04) | 30.1% | 334.67 (62.28) | 100.75 (19.69) |
| Memory problems | No | 3.7% | 106.71 (9.68) | 3.90 (0.37) | 10.4% | 175.66 (11.94) | 18.26 (1.28) |
| Mental | Yes | 6.5% | 166.08 (29.38) | 10.83 (1.98) | 16.5% | 237.80 (21.27) | 39.18 (3.67) |
| Mental | No | 3.2% | 102.96 (11.51) | 3.27 (0.38) | 9.3% | 158.32 (14.06) | 14.69 (1.33) |
| Multiple sclerosis | Yes | 13.4% | 773.82 (283.00) | 103.58 (42.41) | 32.6% | 479.02 (137.99) | 156.35 (46.93) |
| Multiple sclerosis | No | 3.8% | 109.38 (10.65) | 4.13 (0.41) | 10.6% | 176.61 (11.65) | 18.64 (1.26) |
| Musculoskeletal | Yes | 5.6% | 121.60 (13.76) | 6.86 (0.80) | 14.3% | 192.18 (14.42) | 27.44 (2.12) |
| Musculoskeletal | No | 2.0% | 131.13 (27.80) | 2.66 (0.58) | 7.1% | 162.93 (20.21) | 11.58 (1.47) |
| Neurological | Yes | 5.4% | 119.44 (13.40) | 6.49 (0.80) | 15.0% | 237.42 (38.18) | 35.58 (5.90) |
| Neurological | No | 3.6% | 125.31 (15.32) | 4.48 (0.56) | 10.0% | 168.65 (11.08) | 16.83 (1.14) |
| Ophthalmologic | Yes | 7.7% | 115.49 (10.86) | 8.84 (0.88) | 14.4% | 227.84 (26.68) | 32.70 (3.94) |
| Ophthalmologic | No | 2.7% | 131.58 (21.34) | 3.53 (0.59) | 9.6% | 161.89 (11.99) | 15.52 (1.19) |
| Parkinsonism | Yes | 14.5% | 166.68 (54.37) | 24.19 (8.84) | 26.9% | 493.13 (180.53) | 132.49 (50.66) |
| Parkinsonism | No | 3.8% | 123.62 (12.64) | 4.71 (0.50) | 10.6% | 180.06 (11.75) | 19.17 (1.29) |
| Respiratory | Yes | 6.4% | 116.80 (16.87) | 7.43 (1.11) | 16.1% | 239.37 (32.99) | 38.64 (5.49) |
| Respiratory | No | 3.4% | 126.71 (15.84) | 4.27 (0.55) | 9.7% | 164.67 (11.40) | 15.93 (1.14) |
| Stroke | Yes | 15.7% | 191.56 (49.74) | 30.03 (8.43) | 25.3% | 327.51 (56.63) | 83.01 (15.42) |
| Stroke | No | 3.7% | 120.39 (12.90) | 4.44 (0.49) | 10.5% | 177.93 (11.97) | 18.69 (1.29) |
| **Women** |  |  |  |  |  |  |  |
| Bowel incontinence | Yes | 15.8% | 185.28 (58.40) | 29.30 (10.00) | 27.9% | 309.17 (58.59) | 86.24 (17.44) |
| Bowel incontinence | No | 4.4% | 115.95 (15.94) | 5.10 (0.72) | 12.5% | 190.74 (16.07) | 23.93 (2.08) |
| Cancer | Yes | 8.8% | 84.20 (13.29) | 7.42 (1.24) | 19.1% | 213.59 (27.09) | 40.81 (5.38) |
| Cancer | No | 4.0% | 134.56 (20.32) | 5.38 (0.84) | 11.9% | 192.23 (18.28) | 22.88 (2.24) |
| Cardiac | Yes | 12.0% | 120.89 (14.97) | 14.56 (1.95) | 21.2% | 250.86 (30.25) | 53.30 (6.67) |
| Cardiac | No | 3.5% | 121.61 (22.47) | 4.24 (0.80) | 11.6% | 180.66 (18.05) | 20.89 (2.15) |
| Endocrine/Metabolic | Yes | 6.7% | 103.85 (11.26) | 6.99 (0.82) | 16.1% | 249.18 (35.55) | 40.12 (5.90) |
| Endocrine/Metabolic | No | 3.8% | 134.56 (25.66) | 5.10 (1.00) | 11.5% | 165.46 (12.84) | 19.09 (1.55) |
| Gastrointestinal | Yes | 8.1% | 108.13 (15.31) | 8.74 (1.33) | 19.1% | 249.79 (28.58) | 47.64 (5.70) |
| Gastrointestinal | No | 4.0% | 126.80 (20.83) | 5.04 (0.85) | 11.7% | 178.99 (18.41) | 20.85 (2.20) |
| Genitourinary | Yes | 10.8% | 192.36 (49.29) | 20.69 (5.46) | 21.7% | 236.18 (23.18) | 51.35 (5.40) |
| Genitourinary | No | 3.8% | 93.15 (8.60) | 3.55 (0.35) | 11.7% | 186.25 (18.67) | 21.71 (2.24) |
| Hypertension | Yes | 7.5% | 103.56 (10.93) | 7.75 (0.88) | 16.3% | 234.58 (22.34) | 38.27 (3.80) |
| Hypertension | No | 3.4% | 139.20 (28.85) | 4.71 (1.00) | 11.3% | 171.84 (21.16) | 19.50 (2.47) |
| Memory problems | Yes | 17.8% | 420.59 (217.93) | 75.04 (40.43) | 34.8% | 288.86 (51.87) | 100.39 (19.87) |
| Memory problems | No | 4.5% | 103.24 (9.12) | 4.61 (0.43) | 12.6% | 192.76 (16.05) | 24.23 (2.08) |
| Mental | Yes | 7.2% | 173.67 (38.76) | 12.52 (2.88) | 18.1% | 241.46 (21.27) | 43.74 (4.10) |
| Mental | No | 3.9% | 90.25 (8.25) | 3.48 (0.34) | 11.2% | 173.67 (20.83) | 19.53 (2.40) |
| Multiple sclerosis | Yes | 12.4% | 471.27 (170.11) | 58.21 (25.34) | 30.8% | 400.32 (124.70) | 123.39 (40.42) |
| Multiple sclerosis | No | 4.6% | 112.56 (15.02) | 5.17 (0.71) | 12.7% | 192.02 (15.64) | 24.45 (2.05) |
| Musculoskeletal | Yes | 6.7% | 129.38 (19.05) | 8.61 (1.31) | 16.6% | 208.35 (19.46) | 34.54 (3.33) |
| Musculoskeletal | No | 2.2% | 91.54 (16.25) | 2.02 (0.38) | 8.4% | 168.02 (24.50) | 14.07 (2.11) |
| Neurological | Yes | 5.8% | 119.71 (15.79) | 6.90 (1.00) | 16.1% | 244.81 (46.86) | 39.44 (7.78) |
| Neurological | No | 4.4% | 121.90 (19.90) | 5.34 (0.90) | 12.1% | 180.21 (13.33) | 21.77 (1.67) |
| Ophthalmologic | Yes | 9.2% | 112.15 (11.89) | 10.31 (1.17) | 16.8% | 244.81 (37.48) | 41.05 (6.47) |
| Ophthalmologic | No | 3.0% | 131.25 (29.33) | 4.00 (0.91) | 11.5% | 171.62 (13.28) | 19.77 (1.60) |
| Parkinsonism | Yes | 22.7% | 114.64 (38.92) | 26.07 (10.47) | 33.2% | 217.48 (82.41) | 72.31 (31.49) |
| Parkinsonism | No | 4.6% | 121.42 (15.57) | 5.62 (0.74) | 12.9% | 196.51 (15.63) | 25.27 (2.07) |
| Respiratory | Yes | 7.3% | 94.89 (10.71) | 6.97 (0.86) | 18.7% | 260.36 (47.05) | 48.72 (9.08) |
| Respiratory | No | 4.1% | 131.55 (20.93) | 5.38 (0.88) | 11.7% | 174.65 (13.08) | 20.35 (1.58) |
| Stroke | Yes | 16.0% | 166.57 (38.47) | 26.73 (7.17) | 28.1% | 272.37 (44.39) | 76.57 (14.65) |
| Stroke | No | 4.5% | 119.60 (15.94) | 5.43 (0.75) | 12.7% | 194.79 (15.89) | 24.80 (2.09) |
| **Men** |  |  |  |  |  |  |  |
| Bowel incontinence | Yes | 17.4% | 131.42 (38.66) | 22.87 (7.35) | 22.3% | 593.90 (330.51) | 132.44 (76.91) |
| Bowel incontinence | No | 2.8% | 128.32 (22.57) | 3.62 (0.65) | 8.3% | 146.30 (14.53) | 12.11 (1.23) |
| Cancer | Yes | 6.4% | 75.98 (11.84) | 4.86 (0.80) | 13.5% | 226.34 (61.31) | 30.52 (8.46) |
| Cancer | No | 2.5% | 146.35 (28.02) | 3.72 (0.73) | 7.8% | 144.91 (16.44) | 11.25 (1.31) |
| Cardiac | Yes | 7.3% | 128.85 (28.25) | 9.38 (2.11) | 15.1% | 229.95 (41.28) | 34.67 (6.39) |
| Cardiac | No | 2.1% | 128.29 (30.53) | 2.64 (0.65) | 7.0% | 127.58 (17.22) | 8.92 (1.23) |
| Endocrine/Metabolic | Yes | 5.6% | 124.05 (26.88) | 6.95 (1.55) | 11.8% | 175.94 (26.05) | 20.79 (3.18) |
| Endocrine/Metabolic | No | 2.3% | 131.52 (30.40) | 3.01 (0.71) | 7.5% | 153.68 (22.70) | 11.56 (1.74) |
| Gastrointestinal | Yes | 4.6% | 123.36 (23.63) | 5.73 (1.17) | 12.9% | 150.80 (24.30) | 19.40 (3.27) |
| Gastrointestinal | No | 2.8% | 129.69 (25.39) | 3.60 (0.72) | 7.8% | 162.44 (20.94) | 12.75 (1.68) |
| Genitourinary | Yes | 10.4% | 236.90 (82.10) | 24.75 (8.83) | 18.5% | 280.43 (55.84) | 51.98 (10.70) |
| Genitourinary | No | 2.5% | 100.47 (15.81) | 2.54 (0.41) | 7.8% | 142.47 (18.55) | 11.13 (1.48) |
| Hypertension | Yes | 4.5% | 89.78 (15.60) | 4.06 (0.72) | 10.6% | 156.94 (17.81) | 16.70 (1.96) |
| Hypertension | No | 2.2% | 170.86 (40.72) | 3.74 (0.92) | 7.3% | 162.97 (28.22) | 11.86 (2.10) |
| Memory problems | Yes | 13.8% | 338.00 (121.81) | 46.68 (18.13) | 25.5% | 395.83 (128.02) | 101.11 (33.79) |
| Memory problems | No | 2.8% | 112.34 (20.61) | 3.17 (0.60) | 8.2% | 148.70 (17.45) | 12.15 (1.46) |
| Mental | Yes | 5.4% | 149.70 (40.13) | 8.09 (2.27) | 13.8% | 230.04 (48.74) | 31.79 (6.94) |
| Mental | No | 2.6% | 120.49 (24.90) | 3.09 (0.65) | 7.5% | 137.14 (17.05) | 10.25 (1.30) |
| Multiple sclerosis | Yes | 16.3% | 1414.8 (774.22) | 230.19 (143.02) | 37.7% | 658.54 (348.31) | 248.33 (137.08) |
| Multiple sclerosis | No | 3.0% | 104.36 (13.81) | 3.08 (0.42) | 8.3% | 152.67 (17.10) | 12.73 (1.46) |
| Musculoskeletal | Yes | 4.4% | 106.65 (16.54) | 4.66 (0.75) | 11.4% | 162.48 (19.74) | 18.48 (2.31) |
| Musculoskeletal | No | 1.9% | 169.80 (52.41) | 3.19 (1.01) | 6.1% | 157.08 (33.09) | 9.51 (2.04) |
| Neurological | Yes | 4.6% | 118.58 (24.82) | 5.46 (1.29) | 12.1% | 212.59 (53.65) | 25.81 (6.74) |
| Neurological | No | 2.9% | 129.97 (23.97) | 3.71 (0.70) | 8.1% | 153.34 (18.75) | 12.44 (1.55) |
| Ophthalmologic | Yes | 5.6% | 122.80 (22.87) | 6.87 (1.32) | 11.1% | 193.73 (27.48) | 21.56 (3.17) |
| Ophthalmologic | No | 2.3% | 132.00 (31.05) | 3.09 (0.74) | 7.8% | 148.24 (21.97) | 11.51 (1.74) |
| Parkinsonism | Yes | 10.1% | 229.43 (107.27) | 23.18 (12.36) | 23.4% | 702.59 (308.06) | 164.73 (76.04) |
| Parkinsonism | No | 3.0% | 127.13 (21.42) | 3.77 (0.65) | 8.4% | 154.17 (17.54) | 12.92 (1.50) |
| Respiratory | Yes | 5.1% | 158.05 (44.21) | 8.04 (2.30) | 12.8% | 199.29 (33.38) | 25.49 (4.44) |
| Respiratory | No | 2.7% | 119.43 (24.14) | 3.18 (0.66) | 7.7% | 149.97 (20.59) | 11.61 (1.63) |
| Stroke | Yes | 15.4% | 211.04 (82.95) | 32.49 (13.72) | 23.3% | 377.33 (99.96) | 87.82 (24.62) |
| Stroke | No | 2.8% | 121.70 (21.83) | 3.42 (0.63) | 8.2% | 151.06 (17.94) | 12.41 (1.51) |
