# Supplementary material for: Formal and informal care received by middle-aged and older adults with chronic conditions in Canada: CLSA data
Source: PLoS One. 2020 Jul 7;15(7):e0235774. doi: 10.1371/journal.pone.0235774 (PMC7340302; doi:10.1371/journal.pone.0235774)
Supplement: S6 Table — *p value < 0.05. (DOCX) [file pone.0235774.s006.docx]

**S6 Table. Odds ratio of receiving formal/informal care and ratio of expected hours among all study population who received formal/informal care**

| **Parameter** | **Formal care** | | **Informal care** | |
| --- | --- | --- | --- | --- |
|  | **Odds ratio** | **Hours ratio** | **Odds ratio** | **Hours ratio** |
| Bowel incontinence | 1.67 (1.36,2.06)* | 1.10 (0.83,1.45) | 1.25 (1.05,1.48)* | 1.14 (0.89,1.46) |
| Cancer | 1.58 (1.42,1.76)* | 0.73 (0.62,0.86)* | 1.49 (1.38,1.61)* | 1.19 (1.06,1.35)* |
| Cardiac | 1.75 (1.58,1.93)* | 0.97 (0.83,1.13) | 1.57 (1.45,1.68)* | 1.14 (1.02,1.29)* |
| Endocrine/Metabolic | 1.30 (1.18,1.44)* | 1.02 (0.87,1.18) | 1.14 (1.07,1.22)* | 1.10 (0.99,1.23) |
| Gastrointestinal | 1.19 (1.06,1.33)* | 1.08 (0.92,1.28) | 1.24 (1.15,1.34)* | 1.02 (0.91,1.15) |
| Genitourinary | 1.55 (1.38,1.74)* | 1.22 (1.03,1.44)* | 1.40 (1.29,1.53)* | 1.16 (1.02,1.33)* |
| Hypertension | 1.19 (1.08,1.32)* | 0.99 (0.85,1.16) | 1.15 (1.08,1.23)* | 1.15 (1.04,1.28)* |
| Memory problems | 2.19 (1.75,2.74)* | 2.24 (1.67,3.00)* | 1.85 (1.55,2.21)* | 1.86 (1.46,2.36)* |
| Mental | 1.47 (1.32,1.64)* | 1.12 (0.96,1.32) | 1.39 (1.30,1.50)* | 1.43 (1.28,1.60)* |
| Multiple sclerosis | 2.91 (1.96,4.32)* | 8.94 (5.53,14.44)* | 3.25 (2.47,4.27)* | 2.56 (1.80,3.63)* |
| Musculoskeletal | 1.79 (1.60,1.99)* | 1.22 (1.03,1.43)* | 1.73 (1.61,1.85)* | 1.21 (1.09,1.34)* |
| Neurological | 1.09 (0.96,1.23) | 1.29 (1.08,1.55)* | 1.07 (0.99,1.16) | 1.17 (1.03,1.32)* |
| Ophthalmologic | 1.29 (1.16,1.44)* | 1.08 (0.91,1.27) | 1.23 (1.14,1.33)* | 1.10 (0.97,1.25) |
| Parkinsonism | 2.95 (1.88,4.62)* | 1.88 (0.98,3.59) | 2.98 (2.08,4.26)* | 2.38 (1.38,4.11)* |
| Respiratory | 1.28 (1.15,1.43)* | 0.94 (0.80,1.10) | 1.30 (1.21,1.40)* | 1.22 (1.09,1.37)* |
| Stroke | 1.88 (1.51,2.34)* | 1.70 (1.23,2.34)* | 1.78 (1.48,2.13)* | 1.71 (1.29,2.27)* |
| Female | 1.11 (1.00,1.23) | 1.20 (1.03,1.41)* | 1.33 (1.25,1.42)* | 1.41 (1.27,1.57)* |

^*^p value < 0.05
