# Supplementary material for: Formal and informal care received by middle-aged and older adults with chronic conditions in Canada: CLSA data
Source: PLoS One. 2020 Jul 7;15(7):e0235774. doi: 10.1371/journal.pone.0235774 (PMC7340302; doi:10.1371/journal.pone.0235774)
Supplement: S7 Table — aAverage overall expected number of hours assuming all study samples were with a specific condition while keeping all other conditions and characteristics unchanged; bAverage overall expected number of hours assuming all study samples without a specific condition while keeping all other conditions and characteristics unchanged; cThe difference between a and b is the average incremental hours for the specific condition. (DOCX) [file pone.0235774.s007.docx]

**S7 Table. Incremental number of hours of formal care and informal care by chronic conditions**

| **Chronic condition** | **Formal care** | | | **Informal care** | | |
| --- | --- | --- | --- | --- | --- | --- |
|  | **Predicted hours for the condition^a^** | **Predicted hours without the condition^b^** | **Incremental hours per adult^c^** | **Predicted hours for the condition^a^** | **Predicted hours without the condition^b^** | **Incremental hours per adult^c^** |
| **All** |  |  |  |  |  |  |
| Bowel incontinence | 6.50 | 3.95 | 2.55 | 23.60 | 17.64 | 5.96 |
| Cancer | 4.35 | 4.16 | 0.19 | 26.38 | 16.42 | 9.96 |
| Cardiac | 5.44 | 3.58 | 1.86 | 25.18 | 15.67 | 9.50 |
| Endocrine/Metabolic | 4.76 | 3.79 | 0.97 | 20.26 | 16.62 | 3.65 |
| Gastrointestinal | 4.87 | 3.92 | 0.95 | 20.64 | 17.16 | 3.48 |
| Genitourinary | 6.15 | 3.56 | 2.59 | 24.68 | 16.44 | 8.24 |
| Hypertension | 4.46 | 3.90 | 0.55 | 20.60 | 16.02 | 4.57 |
| Memory problems | 14.78 | 3.55 | 11.23 | 48.62 | 16.68 | 31.94 |
| Mental | 5.47 | 3.59 | 1.88 | 26.57 | 14.41 | 12.16 |
| Multiple sclerosis | 69.69 | 3.42 | 66.27 | 97.87 | 17.05 | 80.82 |
| Musculoskeletal | 5.01 | 2.56 | 2.44 | 21.86 | 11.80 | 10.06 |
| Neurological | 5.27 | 3.82 | 1.45 | 20.99 | 17.04 | 3.96 |
| Ophthalmologic | 4.84 | 3.67 | 1.17 | 21.25 | 16.46 | 4.79 |
| Parkinsonism | 17.17 | 4.10 | 13.06 | 88.67 | 17.69 | 70.97 |
| Respiratory | 4.59 | 4.03 | 0.56 | 24.03 | 16.09 | 7.94 |
| Stroke | 10.82 | 3.92 | 6.90 | 44.71 | 17.22 | 27.49 |
| **Women** |  |  |  |  |  |  |
| Bowel incontinence | 6.63 | 5.04 | 1.59 | 31.37 | 24.40 | 6.97 |
| Cancer | 5.04 | 5.29 | -0.26 | 37.39 | 22.41 | 14.98 |
| Cardiac | 7.47 | 4.17 | 3.29 | 30.78 | 23.10 | 7.68 |
| Endocrine/Metabolic | 5.54 | 4.93 | 0.62 | 28.04 | 22.65 | 5.39 |
| Gastrointestinal | 6.34 | 4.73 | 1.61 | 28.39 | 23.52 | 4.87 |
| Genitourinary | 7.26 | 4.47 | 2.79 | 32.28 | 22.83 | 9.45 |
| Hypertension | 5.51 | 4.89 | 0.62 | 27.86 | 22.64 | 5.22 |
| Memory problems | 18.40 | 4.45 | 13.95 | 53.24 | 23.72 | 29.52 |
| Mental | 6.87 | 4.35 | 2.52 | 35.57 | 19.76 | 15.82 |
| Multiple sclerosis | 57.47 | 4.39 | 53.07 | 101.49 | 23.71 | 77.78 |
| Musculoskeletal | 6.18 | 2.81 | 3.38 | 30.25 | 15.17 | 15.08 |
| Neurological | 6.11 | 4.82 | 1.29 | 28.94 | 23.21 | 5.73 |
| Ophthalmologic | 5.90 | 4.59 | 1.31 | 28.91 | 22.69 | 6.22 |
| Parkinsonism | 14.27 | 5.15 | 9.13 | 50.90 | 24.72 | 26.18 |
| Respiratory | 5.32 | 5.15 | 0.18 | 31.14 | 22.61 | 8.52 |
| Stroke | 13.09 | 4.94 | 8.14 | 58.98 | 24.03 | 34.95 |
| **Men** |  |  |  |  |  |  |
| Bowel incontinence | 6.78 | 2.84 | 3.93 | 16.36 | 11.00 | 5.36 |
| Cancer | 3.60 | 2.95 | 0.65 | 16.26 | 10.39 | 5.87 |
| Cardiac | 3.57 | 2.80 | 0.76 | 18.15 | 8.55 | 9.59 |
| Endocrine/Metabolic | 3.87 | 2.72 | 1.15 | 12.50 | 10.69 | 1.81 |
| Gastrointestinal | 3.32 | 3.01 | 0.31 | 12.82 | 10.93 | 1.88 |
| Genitourinary | 4.74 | 2.61 | 2.12 | 17.79 | 10.22 | 7.57 |
| Hypertension | 3.33 | 2.81 | 0.52 | 13.10 | 9.65 | 3.46 |
| Memory problems | 10.82 | 2.69 | 8.13 | 44.78 | 9.79 | 34.99 |
| Mental | 3.96 | 2.81 | 1.15 | 18.44 | 9.07 | 9.36 |
| Multiple sclerosis | 154.60 | 2.42 | 152.17 | 144.71 | 10.77 | 133.94 |
| Musculoskeletal | 3.86 | 2.24 | 1.62 | 13.87 | 8.03 | 5.85 |
| Neurological | 4.89 | 2.81 | 2.09 | 12.70 | 11.03 | 1.67 |
| Ophthalmologic | 3.83 | 2.68 | 1.15 | 13.81 | 10.33 | 3.48 |
| Parkinsonism | 16.92 | 2.97 | 13.95 | 115.48 | 10.63 | 104.86 |
| Respiratory | 3.70 | 2.91 | 0.79 | 17.11 | 9.87 | 7.24 |
| Stroke | 8.75 | 2.75 | 6.00 | 28.34 | 10.49 | 17.85 |

^a^Average overall expected number of hours assuming all study samples were with a specific condition while keeping all other conditions and characteristics unchanged;

^b^Average overall expected number of hours assuming all study samples without a specific condition while keeping all other conditions and characteristics unchanged;

^c^The difference between a and b is the average incremental hours for the specific condition
