# Supplementary material for: Formal and informal care received by middle-aged and older adults with chronic conditions in Canada: CLSA data
Source: PLoS One. 2020 Jul 7;15(7):e0235774. doi: 10.1371/journal.pone.0235774 (PMC7340302; doi:10.1371/journal.pone.0235774)
Supplement: S1 File — (DOCX) [file pone.0235774.s008.docx]

**S1 File. Detailed calculation steps for average marginal effects**

Taking stroke as an example, the steps of calculating the average marginal effect are as follows:

1. Assume all individuals have stroke and keep all their other chronic conditions and covariate values as observed, then
   1. estimate the probability (p_a_) of receiving informal care for each individual using the parameters from the first part model;
   2. estimate the number (x_a_) of informal care hours received for each individual who received informal care using the parameters from the second part model;
   3. calculate the number of informal care received for each individual by p_a_*x_a_ + (1-p_a_)*0 = p_a_*x_a_;
   4. take the average among all individuals (E(A)=E(p_a_*x_a_))
2. Assume all individuals do not have stroke and keep all their other chronic conditions and covariate values as observed, then
   1. estimate the probability (p_b_) of receiving informal care for each individual using the parameters from the first part model;
   2. estimate the number (x_b_) of informal care hours received for each individual who received informal care using the parameters from the second part model;
   3. calculate the number of informal care received for each individual by p_b_*x_b_ + (1-p_b_)*0 = p_b_*x_b_;
   4. take the average among all individuals (E(B)=E(p_b_*x_b_))
3. Calculate the marginal effect of stroke for each individual: p_a_*x_a_ - p_b_*x_b_, and the average marginal effect of stroke is E(p_a_*x_a_ - p_b_*x_b_) = E(A) – E(B).

Please note that the estimated average marginal effect depends on population. Our estimates were based on our entire study population.
