# Supplementary material for: Formal and informal care received by middle-aged and older adults with chronic conditions in Canada: CLSA data
Source: PLoS One. 2020 Jul 7;15(7):e0235774. doi: 10.1371/journal.pone.0235774 (PMC7340302; doi:10.1371/journal.pone.0235774)
Supplement: Attachment — Submitted filename: Response to review comments_May 5 2020.docx [file pone.0235774.s009.docx]

May 5, 2020

*PLOS ONE*

Dear Dr. Andrea Gruneir,

RE: Manuscript PONE-D-20-03193, “Formal and informal care received by middle-aged and older adults with chronic conditions in Canada: CLSA data”

Thank you for your recent letter inviting us to revise and resubmit the above manuscript. In the current version of manuscript, as per your suggestion, we have responded to the reviewers’ comments and have made corresponding changes to the manuscript. Please find our responses below according to the comment number of the reviewers.

We are grateful for the reviewers’ comments and the opportunity to improve our manuscript. We hope the manuscript will be of use to the wider community, and look forward to hearing your response.

Sincerely yours,

Wei Zhang, PhD

School of Population and Public Health, University of British Columbia

Centre for Health Evaluation and Outcome Sciences, St. Paul’s Hospital

588-1081 Burrard Street, Vancouver, BC, Canada V6Z 1Y6

Email: wzhang@cheos.ubc.ca

Tel: +1-604-806-9343

**Please note that page numbers referenced in the below responses are of the clean copy of the manuscript.*

**Journal Requirements:**

When submitting your revision, we need you to address these additional requirements.

1. Please ensure that your manuscript meets PLOS ONE's style requirements, including those for file naming. The PLOS ONE style templates can be found at <https://journals.plos.org/plosone/s/file?id=wjVg/PLOSOne_formatting_sample_main_body.pdf>

and <https://journals.plos.org/plosone/s/file?id=ba62/PLOSOne_formatting_sample_title_authors_affiliations.pdf>

**RESPONSE: We have revised our manuscript according to PLOS ONE’s style requirements.**

2. We note that you have indicated that data from this study are available upon request. PLOS only allows data to be available upon request if there are legal or ethical restrictions on sharing data publicly. For information on unacceptable data access restrictions, please see http://journals.plos.org/plosone/s/data-availability#loc-unacceptable-data-access-restrictions.

In your revised cover letter, please address the following prompts:

a) If there are ethical or legal restrictions on sharing a de-identified data set, please explain them in detail (e.g., data contain potentially identifying or sensitive patient information) and who has imposed them (e.g., an ethics committee). Please also provide contact information for a data access committee, ethics committee, or other institutional body to which data requests may be sent.

b) If there are no restrictions, please upload the minimal anonymized data set necessary to replicate your study findings as either Supporting Information files or to a stable, public repository and provide us with the relevant URLs, DOIs, or accession numbers. Please see http://www.bmj.com/content/340/bmj.c181.long for guidelines on how to de-identify and prepare clinical data for publication. For a list of acceptable repositories, please see http://journals.plos.org/plosone/s/data-availability#loc-recommended-repositories.

We will update your Data Availability statement on your behalf to reflect the information you provide.

**RESPONSE: Please update our Data Availability Statement as: The CLSA data used in our study is third-party data. The CLSA data are available from the Canadian Longitudinal Study on Aging (www.clsa-elcv.ca) for researchers who meet the criteria for access to de-identified CLSA data. We had no special access privileges to the data and other researchers will be able to access the data in the same manner as the authors. The specific data application requirements and process can be found at** [**https://www.clsa-elcv.ca/data-access/data-access-application-process**](https://www.clsa-elcv.ca/data-access/data-access-application-process)**. Please email** [**access@clsa-elcv.ca**](mailto:access@clsa-elcv.ca) **to request a Magnolia user account and other information on CLSA data application.**

2. Your ethics statement must appear in the Methods section of your manuscript. If your ethics statement is written in any section besides the Methods, please move it to the Methods section and delete it from any other section. Please also ensure that your ethics statement is included in your manuscript, as the ethics section of your online submission will not be published alongside your manuscript.

**RESPONSE: We have included ethics statement under the Methods section, “Our study was approved by the University of British Columbia-Providence Health Care Research Ethics Board (Ethics Certificate No. H18-01588)” (lines 106-108 on page 6). Also, please note that under our Acknowledge section on page 24, we acknowledge the CLSA study and add the ethics approval information for the entire CLSA study. This acknowledgement is required by CLSA.**

**Additional Editor Comments:**

First, I would like to thank you for your patience while this manuscript was under review. As you will see, we now have 2 reviews of your manuscript. Overall, both are positive and both reviewers remarked on the potential contribution of this work. You will see, too, though, that both have made some suggestions for clarifications in the manuscript, in particular around the classifications of the chronic conditions and more enhanced discussion of some of the limitations (especially related to the study exclusions on dementia/cognitive impairment). You will also see that one of the reviewers has recommended that you incorporate a gender lens into the work given the overall gendered nature of care provision and differences between women and men in chronic condition profiles. This will require some re-analysis but I would strongly encourage you to consider this and at the very least, to provide more discussion of these issues throughout the manuscript.

**RESPONSE: As suggested, we have clarified the classification of the chronic conditions and enhanced discussion of some of the limitations including the study exclusions on** **dementia/cognitive impairment. We have also re-conducted analysis stratified by sex. Please see our detailed responses to these comments below.**

**Reviewer #1:**

1. classification of chronic conditions: with reference to table S2 you describe that you exclude a particular chronic conditions from other conditions of the same class, if the estimated hours (of formal and informal) care differed greatly. This logic is hardly comprehensible. In case of your classification system in step one "circulatory conditions" you pick high blood pressure / hypertension and re-classify it finally as hypertension. Is it due to the fact that hypertension differs from the other conditions belonging to the same class, because of the low mean hours of at-home care? If this is the case, then you should have picked migraine headaches from the class of "neurological conditions" as well. You identify stroke/ CVA as a stand-alone conditions and exclude it from "circulatory conditions". The counterpart of this condition is osteoporosis within the class of "musculoskeletal conditions". Why did you not chose this conditions?

**RESPONSE: We have clarified the detailed steps of our classification in manuscript (1^st^ paragraph on page 8) and presented the steps in S1-S3 Tables. We made the classification decision based on the total number of hours of formal and informal care after step one (existing frameworks). We identified the chronic conditions with a very large total number of hours of formal and informal care (> 120 hours) and then those with a large difference from the average total number of hours in their respective classification (over ± 30 hours). The difference for migraine headaches from the average total hours of epilepsy and migraine headaches (neurological) was 18.6 hours (S3 Table). The difference for osteoporosis from the average total hours in musculoskeletal was 8.8 hours (S3 Table). Thus, they were not considered as a stand-alone classification.**

2. statistical analysis: you applied a two-part model, which I consider a great idea. Part 2 of the model was a GLM either with a gamma or a log-normal distribution. These decisions depended on the vuong test, AIC, and BIC. Why did your decisions depend on all three criterion? The vuong test is based on the BIC. The AIC might prefer a higher amount of parameters in case of large samples as in your study. Thus, it is in my eyes unnecessary to rely on all of them, especially in cases they contradict each other.

**RESPONSE: As suggested, we have now used AIC as the only criterion (line 171 on page 9).**

3. incremental costs: you apply the same wages for informal care and formal/ professional care. Although you discuss this tricky point in your limitation section, I have to comment on it. You assume that you ensure the consistency between formal care and informal care using a replacement cost approach. This slightly contradicts your statement from the paragraph above, where you state that the hours of formal care may be underestimated and as a consequence the hours of formal and informal care are not comparable. I must admit that indirect costs (in this case productivity losses from a social perspective) are difficult to calculate, because it depends on the point of view. I request the authors to comment on the following two proposals: a.) calculate the indirect costs of informal care using a second approach and refer to it as supplementary material; b.) leave out the calculation of incremental costs, because it does not reinforce the meaning of the manuscript towards its policy implications.

**RESPONSE: We agree that it is difficult to calculate indirect costs and there are different methods. Thank you for providing us with two proposals. We have now chosen to leave out the calculation of incremental costs. All the relevant texts have been deleted.**

**Reviewer #2:**

1. The title is not clear regarding the content of the study. Authors should specify that it is the informal and formal care received (and not provided) for adults with chronic conditions. As worded, it is confusing.

**RESPONSE: We have changed our title into “Formal and informal care received by middle-aged and older adults with chronic conditions in Canada: CLSA data**”.

2. In the summary (as in the results section of the main text) it should be clearly specified over what period of time the hours of care received (and the corresponding cost) were calculated (annual? Weekly?). Likewise, better detail in the methods how the chronic conditions suffered have been classified.

**RESPONSE: As suggested, we have clearly specified over what period of time the hours of care received (i.e. annually) in the Abstract and Results section and added more detailed in the methods how the chronic conditions have been classified.**

3. In the introduction: reference 4 on data on the population receiving informal and / or formal care in Canada is from 2014, are there no more current data?

**RESPONSE: Statistics Canada conducted the same survey, General Social Survey - Caregiving and Care Receiving, every 5 years and the most recent one was done in 2018 (https://www23.statcan.gc.ca/imdb/p2SV.pl?Function=getSurvey&SDDS=4502). The data were just released on January 2020. So far, only a brief summary on demographics have been reported and we have updated the information and the reference 4 (lines 45-47 on page 4). However, only % care receivers aged 65 years and older was reported compared with the reported % for care receivers aged 45 years and older in our previous reference 4. The updated results on formal care and informal care have not been reported yet.**

4. On the other hand, the importance of the study is justified on the grounds that home care reduces the need for other more expensive residential care options. This argument does not take into account the indirect costs incurred by informal caregivers who help patients at home, and responds to a model of caregiver as a resource that is widely discussed.

**RESPONSE: We have clarified that home care reduces health care service utilization such as nursing home, hospital care and physician visits. Although informal care could reduce the direct expenditure from the health sector perspective, the indirect costs incurred by informal caregivers can also be large from a societal perspective. The changes are on lines 57-60 on page 4.**

5. The manuscript does not incorporate a gender analysis, which is fundamental in the subject of home care, and informal care in particular, widely feminized. Likewise, suffering from chronic conditions and disability is more frequent in women, who also have a longer life expectancy. The manuscript should incorporate the differences and inequalities between women and men, from the introduction to the method of analysis (sex is considered as a simple adjustment variable, when it should be stratified by sex), the presentation of the data (the tables should to be presented stratified by sex), and in their discussion.

**RESPONSE: As suggested, we have incorporated the differences and inequalities between male and female in the Introduction (last paragraph on page 5), conducted the analysis stratified by sex (lines 178-179 on page 10) and presented the results, and discussed the findings in the Discussion (1^st^ paragraph on page 20, 2^nd^ paragraph on page 21, and last paragraph on page 22). Please note that sex (female versus male) was asked in the CLSA but we explained it as gender (women versus men) and thus explained the corresponding results by gender (lines 145-147 on page 8).**

6. The methodology should specify which are the categories of formal services considered in the questionnaire, and which the categories for informal care are.

**RESPONSE: We have added the categories of formal services and informal care (lines 119-125 on page 7).**

7. The authors would have to explain how they have analyzed the possible coexistence of different chronic conditions classified into different groups.

**RESPONSE: Individuals can have multiple chronic conditions. We did not categorize individuals into different groups with combinations of chronic conditions. Instead, in our regression models, we have treated each final chronic condition group/classification as a separate dummy variable. Each final classification was coded as a dummy variable if a study participant reported having any one chronic condition within a specific classification (lines 142-143 on page 8). For instance, if an individual had any (or some or all) of** **cataracts, glaucoma, and macular degeneration conditions, then the dummy variable for ophthalmologic** **classification equals 1. If an individual did not have any of the three conditions, then the dummy variable for ophthalmologic** **classification equals 0. This implies that, given all other conditions and covariates in the same, the impact of cataracts + glaucoma + macular degeneration on receiving formal/informal care was the same as cataract alone in our regression analyses. However, if an individual had both cataracts and stroke, then dummy variable for ophthalmologic** **classification and the dummy variable for stroke classification are both equal to 1. The impact of cataracts + stroke on receiving formal/informal care would be the sum of the impacts of ophthalmologic and stroke (in log scale) in each part of our regression models, given all other conditions and covariates values unchanged. Since both parts of our two-part model are generalized linear models, the coefficient of the dummy variable for each chronic condition classification from each part could not be interpreted as the same as in a linear model. Therefore, we reported the average marginal effects of chronic condition classifications on formal/informal care hours received among our study population, i.e., the average of marginal effect of each individual in our samples. Detailed calculation steps for average marginal effects are presented in S1 File.**

8. I consider it important, as I said before, that the data be analyzed and presented by sex. Likewise, in Table 1 the formal and informal care time received should be presented by age group.

**RESPONSE: We have analyzed and presented the data by sex. We have also presented the number of formal care hours and number of informal care hours by age group in S4 Table (a table showing all characteristics of study population). Table 1 in the main manuscript only presents some of the characteristics due to the space limit.**

9. As possible limitations, the following should also be discussed:
- Possible limitation of excluding cognitively impaired persons from the study population (care time is underestimated) **(Done, lines 358-363 on page 21)**
- Possible memory bias when asking about the care time received during the previous 12 months, in which the care needs may have been highly variable. **(Done, lines 373-375 on page 22)**
- Possible differences with other studies that ask about the time dedicated to caregivers, instead of people who need care. The perception of time spent can be very different. **(Done, lines 375-377 on page 22)**

**RESPONSE: As suggested, we have discussed all of the limitations in our Discussion section. We have indicated the location of the changes above.**

10. The authors should incorporate into the discussion some implications for the models of substitution or complementarity between formal and informal care.

**RESPONSE: We have discussed the implications of the relationship between formal and informal care. In our study, the four chronic conditions, multiple sclerosis, memory problems, Parkinsonism, and stroke, have consistently been associated with more formal care and more informal care among both women and men, which might suggest a complementary relationship between formal and informal care. However, a future study is required to further investigate the relationship and whether the relationship varies by chronic conditions or functional impairment level (the last paragraph on page 22 and the 1^st^ paragraph on page 23).**

11. Likewise, the implications for gender equality of the study results.

**RESPONSE: We have added the implications for gender inequality of our study results. Multiple sclerosis, Parkinsonism, memory problems, and stroke were the chronic conditions requiring the most formal and informal care among both women and men. Although women overall received more formal and informal care than men, the incremental impact magnitudes of certain chronic conditions (e.g., multiple sclerosis and Parkinsonism) on formal and informal care received by men could be larger than women (1^st^ paragraph on page 20). It is essential for policy makers to recognize the demand for formal and informal care by gender in an aging population. Our study findings can help to prioritize resource allocation for chronic conditions by gender while addressing the increasing demand for formal and informal care in aging populations (the last paragraph on page 22 and the 1^st^ paragraph on page 23).**
